# Comparison of intravenous butorphanol vs. tramadol for post-spinal anesthesia shivering: a meta-analysis and systematic review

**DOI:** 10.3389/fmed.2023.1271664

**Published:** 2023-12-05

**Authors:** Ji-Xiang Wan, Xue-Chao Li, Si-Si Zeng, Yu-Qian Li, Fang-Jun Wang

**Affiliations:** Department of Anesthesiology, Affiliated Hospital of North Sichuan Medical College, Nanchong, Sichuan, China

**Keywords:** butorphanol, tramadol, spinal anesthesia, shivering, meta-analysis

## Abstract

**Background:**

Patients often experience shivering after spinal anesthesia. In recent years, more and more studies have compared the efficacy and side effects of intravenous butorphanol and tramadol in the treatment of shivering after spinal anesthesia. Therefore, we conducted a MATE analysis and systematic review to compare the efficacy and side effects of butorphanol vs. tramadol in the treatment of shivering after spinal anesthesia.

**Methods:**

PubMed, Cochrane Library, and Embase databases were searched for randomized controlled trials (RCTs) from inception to 30 December 2022, comparing the effects of butorphanol vs. tramadol for the control of shivering after spinal anesthesia. Data assessment and collection were analyzed using the Review Manager 5.4 software.

**Results:**

Five randomized controlled trials involving 302 adult patients were included in this meta-analysis. The results showed that butorphanol has a shorter time to cease shivering (standardized mean difference (SMD) = −0.53; 95% confidence interval (CI) [−0.89, −0.17], *P* = 0.004, *I*^2^ = 0%), a higher rate of cessation of shivering within 1 min after administering the study drugs (relative risk (RR), 1.69; 95% CI [1.15,2.48], *P* = 0.008, *I*^2^ = 0%), and higher incidences of sedation (RR, 2.98; 95% CI [2.11, 4.21], *P* <0.00001, *I*^2^ = 0%), compared with tramadol.

**Conclusion:**

In the treatment of shivering after spinal anesthesia, butorphanol has a shorter onset time and a higher rate of cessation of shivering within 1 min after the study drugs were administered than tramadol. Therefore, butorphanol is superior to tramadol in the treatment of shivering after spinal anesthesia.

## Introduction

Spinal anesthesia is widely used in lower abdominal or lower limb surgeries. Shivering is a common complication of spinal anesthesia, with an incidence of approximately 70% (1, 2). Neuraxial (spinal and epidural) anesthesia inhibits vasoconstriction and produces vasodilation, which leads to a rapid loss of heat by redistribution from the core to the periphery. Therefore, the threshold of shivering is reduced (3, 4). There are many reasons for a drop in core temperature, including mental stress, the cold environment of operating rooms, and cold infusion fluids (5, 6). Shivering interferes with the monitoring of vital signs such as pulse rate, blood pressure (BP), and electrocardiography (ECG) (4, 7, 8). It may cause increased wound pain and delayed wound healing, which can prolong the patient's hospital stay (9, 10) and increase intraocular pressure and intracranial pressure (11, 12). Most importantly, it can increase oxygen consumption by 200–600%, and at the same time, increase the production of carbon dioxide linearly (13), leading to hypoxemia. In patients with coronary artery disease, shivering could further compromise myocardial function (14). Therefore, we must promptly control post-anesthetic shivering.

Currently, there are pharmacological and non-pharmacological methods for controlling shivering. The non-pharmacological methods are performed by external heating, such as the use of heaters, blankets, and infusion of warm fluids (15–17). However, some recent studies have shown that non-pharmacological methods, including forced air and warmed fluid, have no significant effect on preventing shivering (18, 19). Therefore, pharmacotherapy is still the main method to control shivering at present.

In the past, tramadol has been widely used in the treatment of shivering after spinal anesthesia (20, 21). It mainly inhibits the synaptic reuptake of norepinephrine and 5-HT (hydroxytryptamine), increasing their concentration outside the neurons, thereby increasing the activity of 5-HT and norepinephrine, and then regulates the monoamine downward inhibitory pathway, resulting in an anti-shivering effect (22–24). However, the main side effects of tramadol are nausea and vomiting (25, 26), which make the patient quite uncomfortable. Currently, some studies have shown that butorphanol has anti-corrosion effects (27). Butorphanol is an opioid receptor agonist-antagonist; it exerts analgesic and anti-shivering effects by activating the K receptor while also partially antagonizing the μ opioid receptor. As a result, it has a minimal impact on respiration and circulation, with only minor adverse reactions (27–29).

The purpose of this study is to conduct a systematic review and meta-analysis to compare the effective rate, the time of onset of action, and the side effects of butorphanol vs. tramadol in the treatment of shivering after spinal anesthesia.

## Methods

This systematic review is based on the Preferred Reporting Items for Systematic Reviews and Meta-Analysis (PRISMA) guidelines for the preparation of the statement of the review. The authors registered the protocol in the International Prospective Register of Systematic Reviews (Registration Number: CRD42022349679; available at https://www.crd.york.ac.uk/prospero/#myprospero).

### Electronic search

A systematic literature search was conducted on the PubMed, Cochrane Library, and Embase databases. Each database was searched by two investigators separately. The databases were searched from inception to 30 December 2022. The subject headings used for searching included tramadol, butorphanol, spinal anesthesia, shivering, and combinations of these without limits. Furthermore, the researchers scanned the references to relative articles to find further studies. An example illustration of the search strategy used for PubMed is shown in Appendices 1 and 2 (Supplementary material).

### Study selection and data extraction

The inclusion criteria for selecting studies were as follows: (1) patients who underwent surgery under spinal anesthesia; (2) the experimental group was given intravenous butorphanol injection, and the control group was given intravenous tramadol; (3) the study was a randomized controlled trial (RCT); (4) patients of American Society of Anesthesiologists Physical Status (ASA) I–III; (5) adult patients.

The exclusion criteria were as follows: (1) retrospective studies, non-randomized and/or randomized literature with incorrect methods; (2) animal studies; (3) case reports. The intensity of shivering was graded on the following scale (30, 31): 0 = no shivering, 1 = no visible muscular activity, but one or more of piloerection, peripheral vasoconstriction, or peripheral (other causes excluded); 2 = visible muscular activity confined to one muscle group; 3 = visible muscular activity in more than one muscle group; 4 = violent muscular activity involving the entire body.

After the studies were selected, two investigators (J-XW and X-CL) independently determined the following primary outcomes and secondary outcomes of shivering treatment (Table 1).

**Table 1:** Characteristics of the included studies.

**References**	**Date**	**Sample size B/T**	**Patient characteristics; surgical setting**	**Dosage**	**Definition of shivering**		**Outcomes measures**
Palan and Agrawal (32)	2017	50/50	20–50 years, ASA: I–II; lower abdominal, urogenital, orthopedic, and gynecological surgeries	Butorphanol 1 mg, tramadol 50 mg	Grades 2 to 4	15–20 mg of 0.5% bupivacaine (hyperbaric)	①③④⑤⑥⑦
Maheshwari et al. (33)	2008	25/25	20–40 years, ASA: I–II	Butorphanol 0.02 mg/kg, tramadol 1 mg/kg	No mention	No mention	①③④⑤⑥⑦
Joshi et al. (34)	2013	15/13	18–60 years, ASA: I–II; elective surgery	Butorphanol 0.03 mg/kg, tramadol 1 mg/kg	Grade 2 or 3	0.5% bupivacaine 3–4 ml	①③④⑤⑥⑦
Bansal and Jain (35)	2011	30/30	18–65 years, ASA: I–III; urological, inguinal, and lower limb surgeries	Butorphanol 1 mg, tramadol 50 mg	Grade 2 or 3	Bupivacaine (0.5%, heavy) in a dose of 3.2–3.5 ml	①②④⑤⑥⑦
Keerthi and Kamath (36)	2017	32/32	18–60 years, ASA: I–II; elective lower abdominal and lower limb surgeries	Butorphanol 0.01 mg/kg, tramadol 0.5 mg/kg	Grades 2 to 4	0.5% hyperbaric bupivacaine 2.8–3 ml	①②④⑤⑥⑦

ASA, American Society of Anesthesiologists. : The effective rate of shivering treatment, : Time to cease shivering, : Time taken to stop shivering after giving study drugs, : Recurrent rate of shivering, : The incidence of nausea, : The incidence of vomiting, : The incidence of sedation.

Primary outcomes:

1. Effective rate of shivering treatment: the number of patients who stopped shivering after treatment.
2. Time to cease shivering: the time from administration of intravenous butorphanol or tramadol to the end of the shivering.
3. The rate of cessation of shivering within a certain period of time after giving study drugs: the number of patients who stopped shivering within a certain period of time after taking the study drugs.
4. Recurrent rate of shivering: the number of patients with a recurrence of chills after treatment.

Secondary outcomes:

1. The incidence of nausea.
2. The incidence of vomiting.
3. The incidence of sedation.

### Selection of studies and quality assessment

First, the titles and abstracts of all retrieved articles were screened, and the full text of the articles that could be included was screened independently by two reviewers (J-XW and S-SZ). The discrepancies were resolved through discussion.

Two investigators (J-XW and Y-QL) independently assessed the risk of bias of the included RCTs based on the Cochrane Manual v5.0.2.10 (37). Each of the following risk-of-bias items was classified as “high risk of bias,” “uncertain risk of bias,” or “low risk of bias”: random sequence generation, allocation concealment, blinding of participants and personnel, blinding of outcome assessment, incomplete outcome data, selective reporting, and other biases. Disputes were resolved by discussion, and if necessary, a third researcher helped make a decision.

### Statistical analysis

Review Manager 5.4 (Cochrane Collaboration, Copenhagen, Denmark) was used to perform all the statistical analyses. For dichotomous data, the Mantel-Haenszel (M-H) method was used to calculate the risk ratio (RR) with a 95% confidence interval (CI). The standardized mean difference was used for continuous variables. The heterogeneity analysis was conducted using the chi-square test, and the heterogeneity of the included studies was assessed using *I*^2^. When the *I*^2^-values were < 40%, 40–60%, and >60%, the heterogeneity levels corresponded to low, medium, and high (38), respectively. Due to the anticipated heterogeneity in this study, a random-effects model was employed. Sensitivity analyses were performed by removing one study at a time and combining the other studies to assess whether a single study significantly affected the pooled results as well as to find the potential causes of the heterogeneity.

## Results

**Figure 1:**
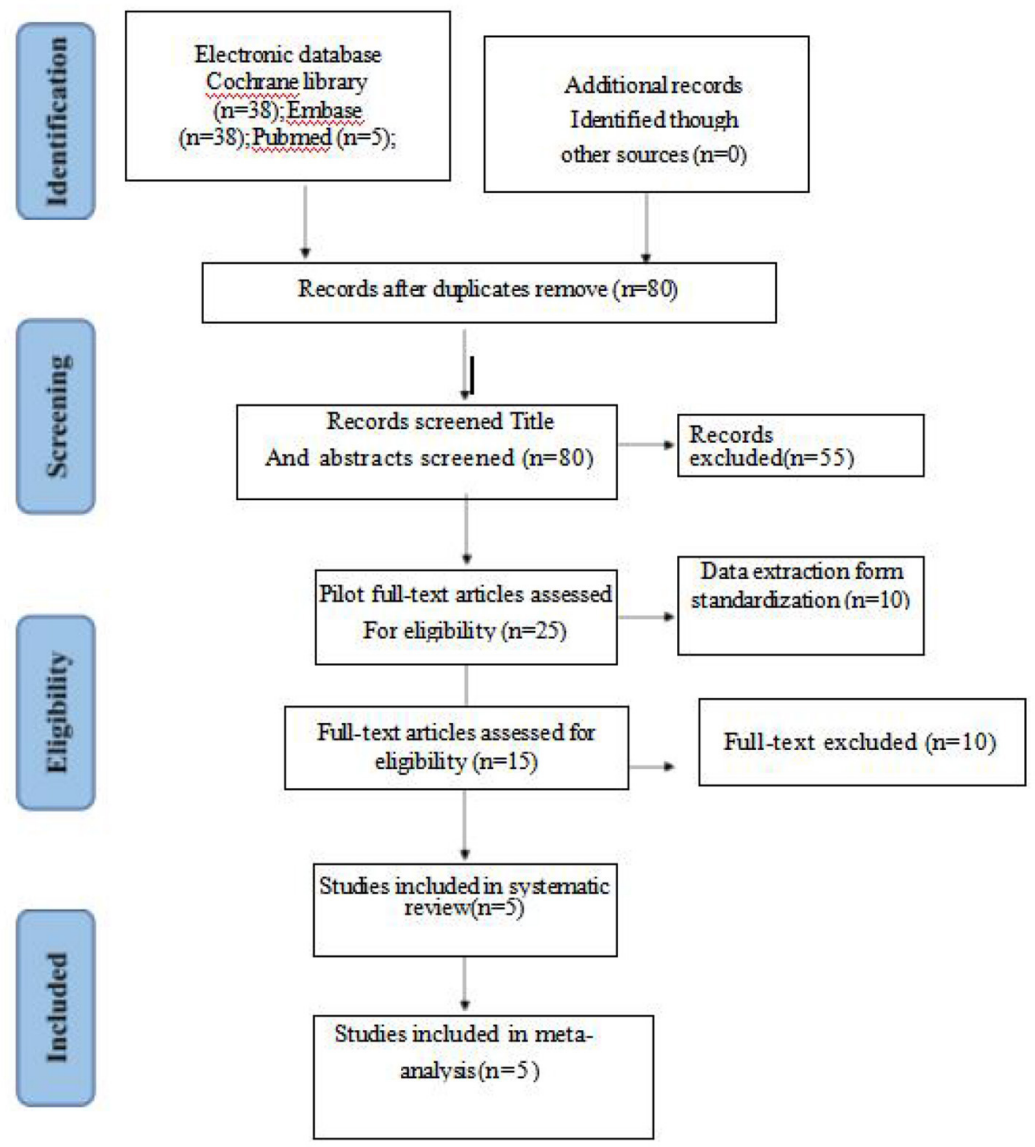
Flowchart for article selection in the meta-analysis.

The research flowchart is shown in Figure 1. We have identified five randomized controlled trials, including 302 patients (152 received butorphanol and 150 received tramadol).

### Study selection

In the five selected studies, two (32, 33) compared butorphanol with tramadol, one (35) compared butorphanol with tramadol and clonidine, one (34) compared butorphanol with tramadol and ondansetron, and the remaining one (36) compared butorphanol with tramadol and dexmedetomidine. However, this meta-analysis only compares butorphanol and tramadol, ignoring clonidine, ondansetron, and dexmedetomidine.

### Study characteristics

The characteristics of the randomized controlled trials (RCTs) are listed in Table 1.

### Study risk of bias

**Figure 2:**
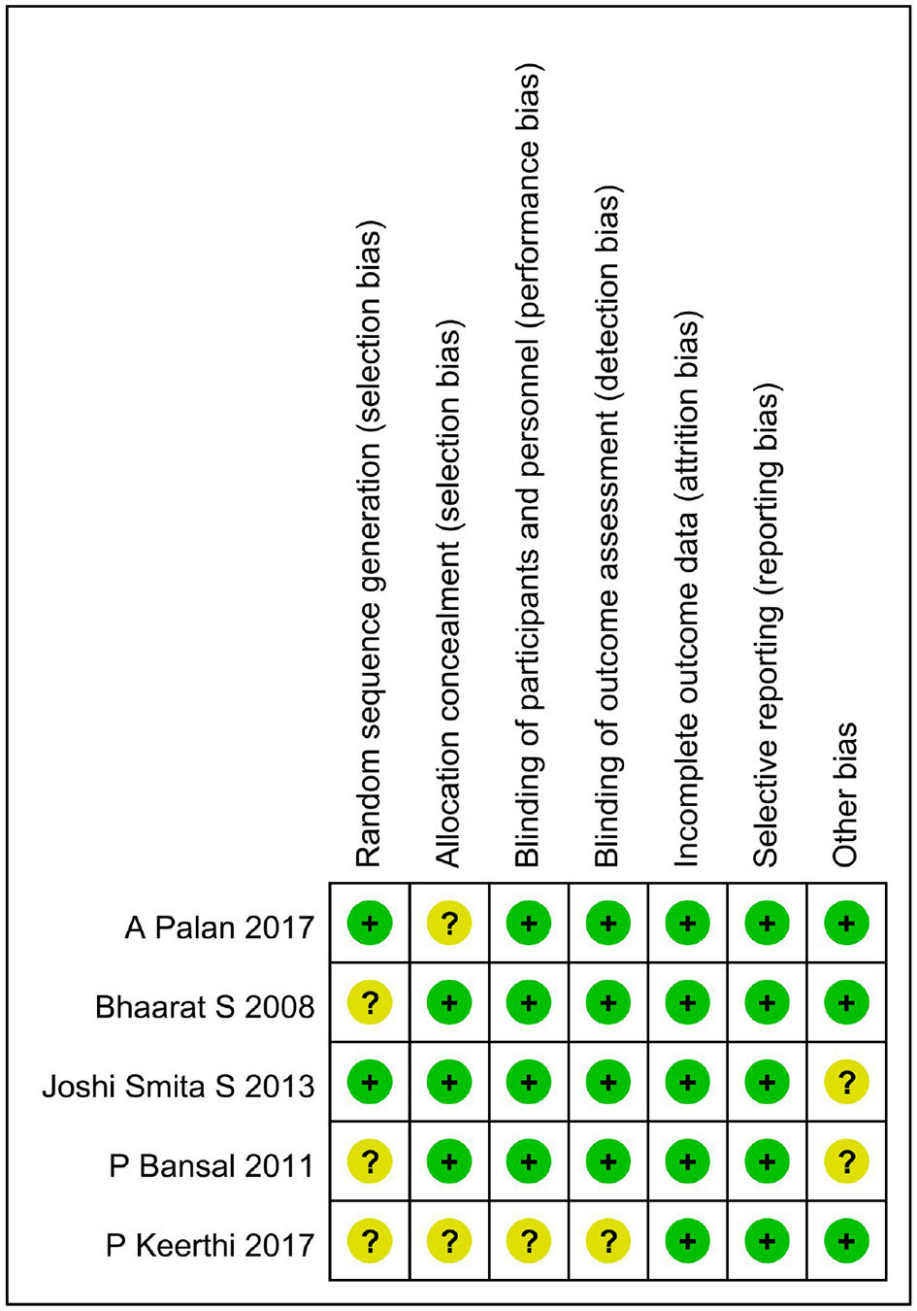
The risk of bias assessment of the included studies. +, low risk of bias; ?, unclear risk of bias; –, high risk of bias.

The summary risk of bias for the five selected studies is quite low, as shown in Figure 2.

### Primary outcome

#### The effective rate of shivering treatment

**Figure 3:**
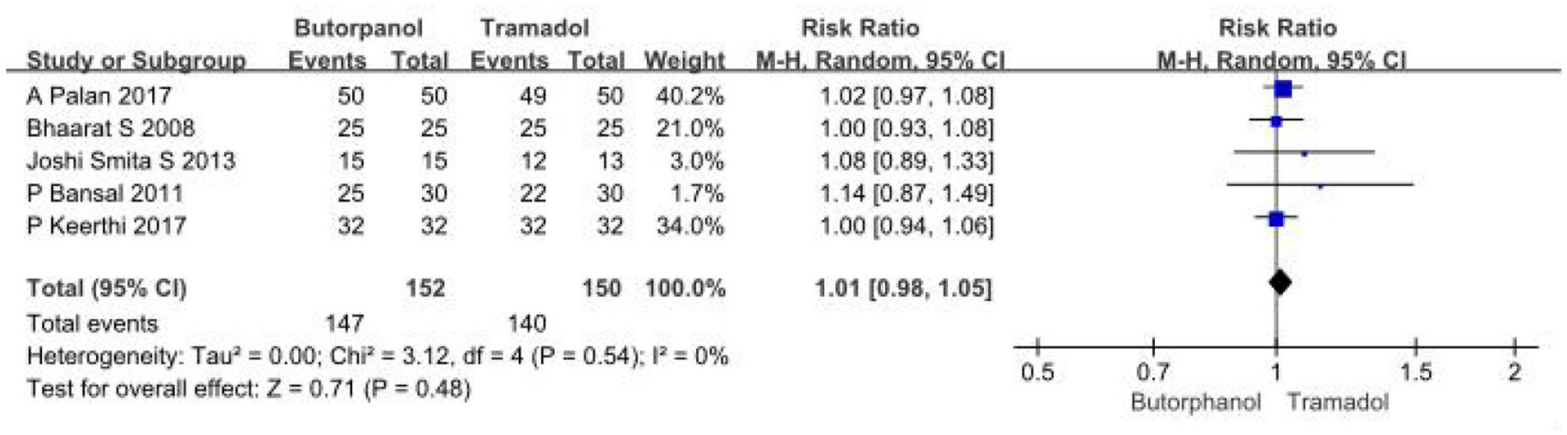
Forest plots of the effective rate of shivering treatment comparing butorphanol with tramadol. CI, confidence interval; M-H, Mantel-Haenszel.

A total of 5 studies (32–36) involving 302 patients directly compared the effective rate of butorphanol and tramadol for shivering after spinal anesthesia, and all data were available for collection (Figure 3). The effective rate of shivering treatment decreased from 97% in the butorphanol group to 93% in the tramadol group (RR, 1.01; 95% CI [0.98, 1.05], *P* = 0.48, *I*^2^ = 0%). The result showed no significant difference between the butorphanol group and the tramadol group.

#### Time to cease shivering (min)

**Figure 4:**
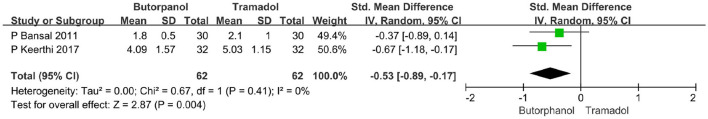
Forest plots of time to cease shivering in minutes comparing butorphanol with tramadol. CI, confidence interval; SMD, standardized mean difference.

A total of 2 studies (35, 36) reported the time to cease shivering, and data from 2 studies with 124 patients were available for collection (Figure 4). The result showed that butorphanol was associated with a shorter time to stop shivering than tramadol (SMD = −0.53; 95% CI [−0.89, −0.17], *P* = 0.004, *I*^2^ = 0%).

#### The rate of cessation of shivering within a certain period of time after giving the study drugs

**Figure 5:**
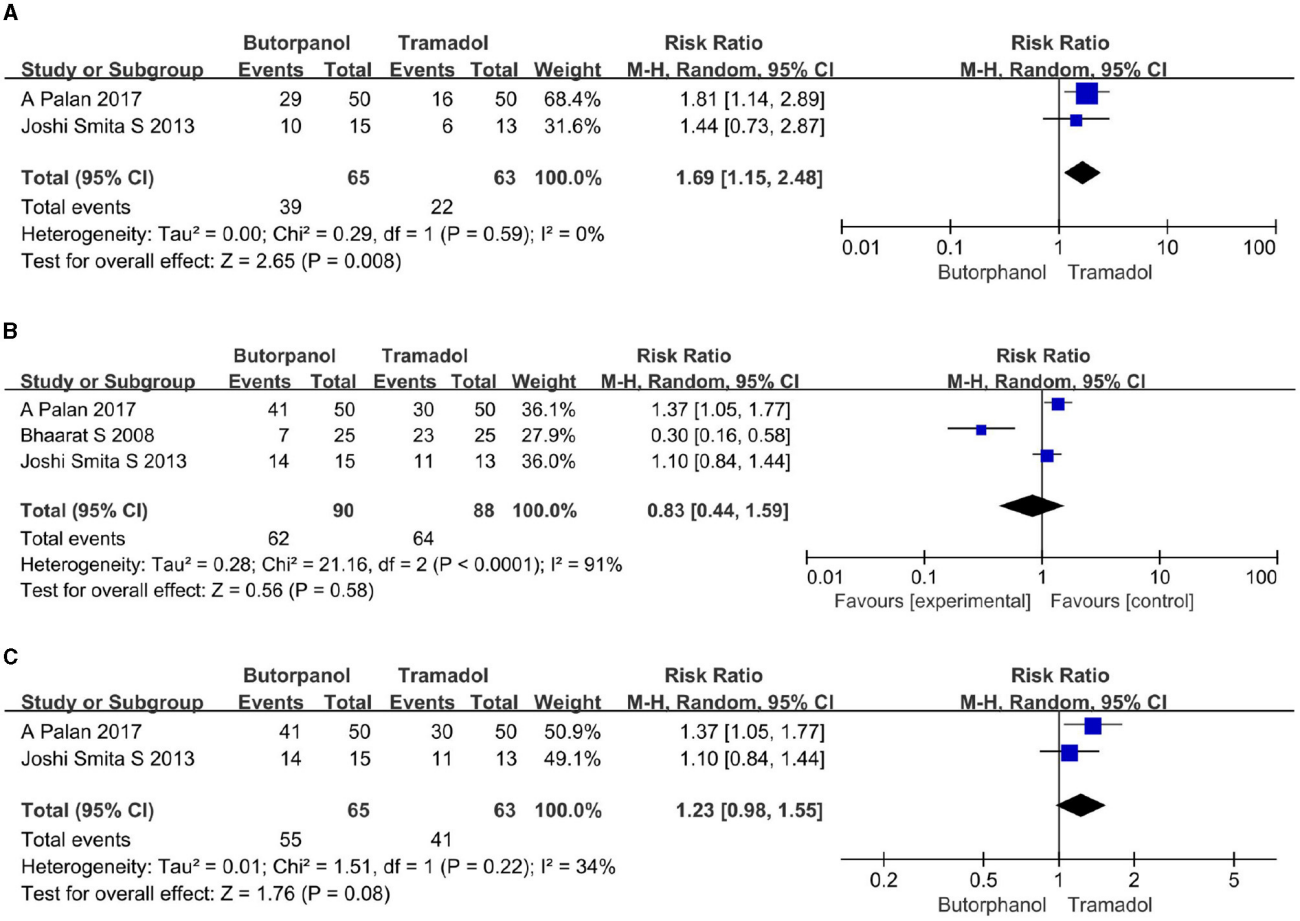
**(A)** Forest plots of time taken to stop shivering within 1 min comparing butorphanol with tramadol. **(B)** Forest plots of time taken to stop shivering within 3 min comparing butorphanol with tramadol. **(C)** Forest plots of time taken to stop shivering within 3 min after sensitivity analysis comparing butorphanol with tramadol. CI, confidence interval; M-H, Mantel-Haenszel.

A total of two studies (32, 34) reported the rate of cessation of shivering within 1 min after giving the study drugs. Data from two studies with 128 patients were available for collection (Figure 5A). The rate of cessation of shivering within 1 min after giving the study drugs decreased from 60.00% in the butorphanol group to 35% in the tramadol group (RR, 1.69; 95% CI [1.15, 2.48]; *P* = 0.008, *I*^2^ = 0%).

A total of three studies (32–34) reported the rate of cessation of shivering within 3 min after giving the study drugs. Data from 3 studies with 178 patients were available for collection. The rate of cessation of shivering within 3 min after giving the study drugs increased from 69% in the butorphanol group to 73% in the tramadol group (Figure 5B), but there was not enough for a statistically significant difference (RR, 0.83; 95% CI [0.44, 1.59]; *P* = 0.58, *I*^2^ = 91%). Sensitivity analysis showed that when the study of Maheshwari et al. (33) was removed (Figure 5C), the heterogeneity was significantly reduced (RR, 1.23; 95% CI [0.98, 1.55]; *P* = 0.08, *I*^2^ = 34%). This shows that the study conducted by Maheshwari et al. (33) is the primary source of heterogeneity, and the result is unstable.

#### Recurrent rate of shivering

**Figure 6:**
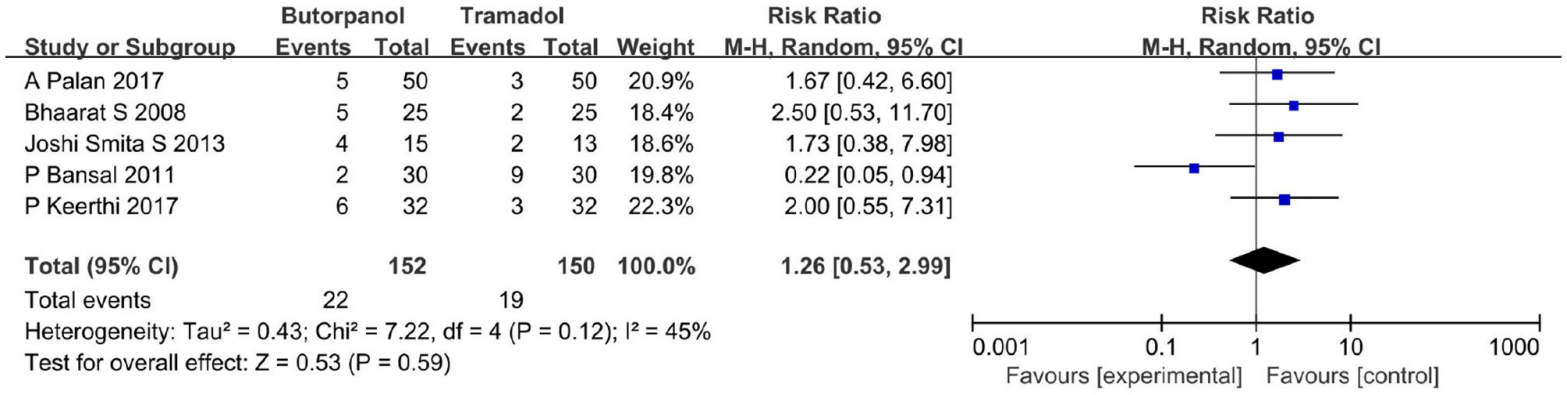
Forest plots of recurrent rates of shivering comparing butorphanol with tramadol. CI, confidence interval; M-H, Mantel-Haenszel.

A total of five studies (32–36) reported the recurrent rate of shivering. Data from five studies with 302 patients were available for collection (Figure 6). The recurrent rate of shivering decreased from 14% in the butorphanol group to 13% in the tramadol group (RR, 1.26; 95% CI [0.53, 2.99], *P* = 0.59, *I*^2^ = 45%). There was no significant difference between the butorphanol group and the tramadol group.

### Secondary outcome

#### The incidence of nausea

**Figure 7:**
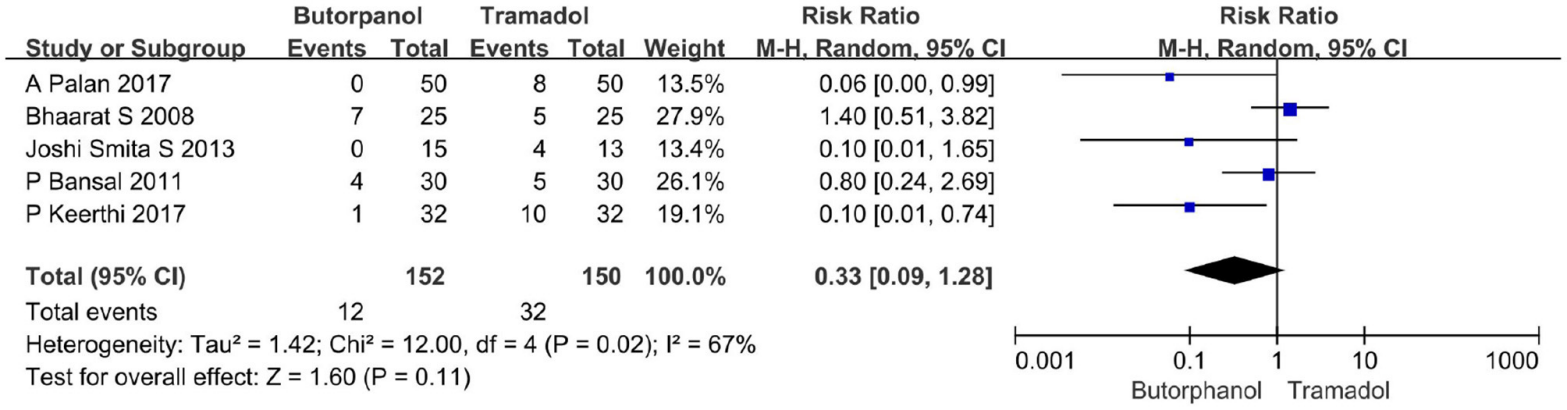
Forest plots of the incidence of nausea comparing butorphanol with tramadol. CI, confidence interval; M-H, Mantel-Haenszel.

A total of five studies (32–36) reported the incidence of nausea. Data from five studies with 302 patients were available for collection (Figure 7). The incidence of nausea increased from 8% in the butorphanol group to 21% in the tramadol group (RR, 0.33; 95% CI [0.09, 1.28], *P* = 0.11, *I*^2^ = 67%). The result did not reveal a significant difference between the butorphanol group and the tramadol group. The sensitivity analysis was conducted, and it was determined that the pooled analysis result remained stable and the heterogeneity was still high.

#### The incidence of vomiting

**Figure 8:**
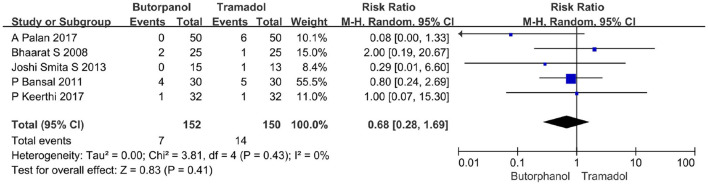
Forest plots of the incidence of vomiting comparing butorphanol with tramadol. CI, confidence interval; M-H, Mantel-Haenszel.

A total of five studies (32–36) reported the incidence of vomiting. Data from five studies with 302 patients were available for collection (Figure 8). The incidence of vomiting increased from 4.61% in the butorphanol group to 9% in the tramadol group (RR, 0.68; 95% CI [0.28, 1.69], *P* = 0.41, *I*^2^ = 0%). There was no significant difference between the butorphanol group and the tramadol group.

#### The incidence of sedation

**Figure 9:**
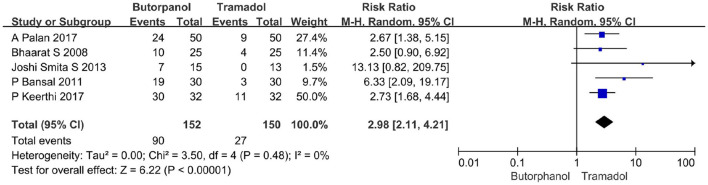
Forest plots of the incidence of sedation comparing butorphanol with tramadol. CI, confidence interval; M-H, Mantel-Haenszel.

A total of five studies (32–36) reported the incidence of sedation. We defined it as being lethargic and responding to speech or physical stimuli. Data from 5 studies with 302 patients were available for collection (Figure 9). The incidence of sedation decreased from 59% in the butorphanol group to 18% in the tramadol group (RR, 2.98; 95% CI [2.11, 4.21], *P* < 0.00001, *I*^2^ = 0%).

## Discussion

In this systematic review and meta-analysis, we compare the efficacy of intravenous butorphanol vs. tramadol for the treatment of chills after spinal anesthesia. Compared with tramadol, butorphanol is associated with a shorter onset time of action, a higher rate of cessation of shivering within 1 min after administering the study drugs, and higher incidences of sedation. Therefore, butorphanol is superior to tramadol in the treatment of chills after spinal anesthesia.

In this meta-analysis, butorphanol has a higher rate of cessation of shivering within 3 min after the study drugs were administered and a lower incidence of nausea than tramadol. However, these outcomes are highly heterogeneous, which may be related to the following factors: (1) The inclusion criteria for the degree of shivering were different among the included studies; two studies (32, 36) used the wrench scale of shivering (30, 31) to grade the intensity of shivering from 0 to 4, and only patients who developed either 2 or 4 shivering were considered for treatment. In two studies (27, 29), the intensity of shivering was scored on a scale of 0–3 (39), and only patients who developed chills of grade 2 or 3 during the perioperative period were treated on an intention-to-treat basis. (2) One study (33) did not mention the type and dosage of local anesthetics for spinal anesthesia, and the dosage of local anesthetics for spinal anesthesia was different among the remaining studies. (3) The type and duration of surgeries were different.

In our study, we found that the butorphanol group (97%) and the tramadol group (93%) were equally effective in controlling shivering, but there was no significant difference (*P* > 0.05). Maheshwari et al. (33) also found similar results that 100% of patients in the butorphanol group and 100% of patients in the tramadol group were relieved of shivering. This suggests that both butorphanol and tramadol can effectively treat shivering after spinal anesthesia; there is no statistically significant difference in the effective rate of shivering treatment. We also found that the time to cease shivering was significantly shorter with the butorphanol group than with the tramadol group (*P* = 0.004). These findings were similar to those of other investigators like Bansal and Jain (35) and Keerthi and Kamath (36), Bansal and Jain (35) observed that butorphanol (1.8 ± 0.5 min) acted faster than tramadol (2.1 ± 1.0 min) to cease shivering after spinal anesthesia, and Keerthi and Kamath (32) found that the time to cease shivering was quite less with butorphanol (4.09 ± 1.57 min) than with tramadol (5.03 ± 1.15 min). Therefore, the time to cease shivering in the butorphanol group is shorter than that in the tramadol group. In the present study, we found that the recurrent rate of shivering was higher in the butorphanol group (14%) than in the tramadol group (13%), but the difference was not statistically significant. Keerthi and Kamath (36) observed a higher rate of recurrence with butorphanol (18%) compared with tramadol (9%). In this study, all patients who received the study drugs had a reappearance of shivering after 20 min of treatment, which suggested that additional anti-shivering drugs were required after 20 min of treatment with butorphanol or tramadol.

In our study, the incidence of nausea was significantly lower with butorphanol (8%) than with tramadol (23%). The results were comparable with studies done by Joshi et al. (34). Four patients (31%) in the tramadol group had nausea as compared to none in the butorphanol group. Contrary to our results, Maheshwari et al. (33) found a higher incidence of nausea with butorphanol (28%) compared with tramadol (20%). This may be due to the small sample size of the trial by Maheshwari et al. Therefore, randomized controlled trials with larger samples are needed for further validation. We also found that butorphanol (59%) had a higher incidence of sedation as compared to tramadol (18%), and this was comparable to observations made by Bansal and Jain (35). This suggests that butorphanol has a higher incidence of sedation in the treatment of shivering after spinal anesthesia compared to tramadol, which is due to the sedative effect of butorphanol by activating K receptors (27, 29). Since operations were performed under spinal anesthesia, the higher incidence of sedation was not only conducive to intraoperative management but also conducive to the surgeon's intraoperative operation and could provide good comfort for the patient. In addition, Keerthi and Kamath (36) observed that at 5 min after the administration of the drug, all cases in the butorphanol group stopped shivering, while 50% of cases in the tramadol group still had grade 1 shivering. There was a significant decrease in shivering grades in the butorphanol group. However, there are few relevant studies at present, and a large sample study is needed in the future.

Most importantly, butorphanol and tramadol were safe for the treatment of chills because there were no patients with excessive sedation (excessive sedation was defined as unresponsiveness to sound or tactile stimuli) in all the included studies. Although one study (36) reported that there were two cases of respiratory depression in the butorphanol group, none of them had hypoxemia, probably because all the patients were supplemented with oxygen at the onset of shivering.

There are a few limitations to our meta-analysis. First, due to the different anesthetic drugs, the patient's physical condition in the American Society of ASA was different; there was significant heterogeneity. Second, many details about the sedation level of each patient at the same time point could not be extracted due to the inconsistency of the scale of evaluating sedation and the different times of evaluating sedation. Third, the sample size is relatively small, proportional to the burden of this perioperative problem. In addition, we have not conducted a dose-response study for a single drug, which can describe its anti-shivering properties and the corresponding increase in side effects. In the future, we can do some studies in this area or compare the efficacy of combination drugs to control chills.

In general, compared with tramadol, we found that butorphanol has a shorter onset time and, at the same time, a higher rate of cessation of shivering within 1 min after giving study drugs. Therefore, butorphanol is superior to tramadol in the treatment of shivering after spinal anesthesia.

## Author contributions

J-XW: Conceptualization, Data curation, Investigation, Methodology, Writing—original draft, Writing—review & editing. X-CL: Conceptualization, Data curation, Investigation, Methodology, Writing—original draft, Formal analysis, Project administration. S-SZ: Formal analysis, Resources, Software, Supervision, Writing—review & editing. Y-QL: Conceptualization, Data curation, Formal analysis, Funding acquisition, Project administration, Resources, Supervision, Writing—review & editing. F-JW: Conceptualization, Formal analysis, Investigation, Methodology, Project administration, Software, Visualization, Writing—original draft, Writing—review & editing.
